# Frequency of Intrusions and Appraisal of Related Distress After Analogue Trauma: A Comparative Ecological Momentary Assessment Methods Study

**DOI:** 10.1007/s10608-018-9941-6

**Published:** 2018-07-10

**Authors:** Julina A. Rattel, Lisa M. Grünberger, Julia Reichenberger, Michael Liedlgruber, Stephan F. Miedl, Jens Blechert, Frank H. Wilhelm

**Affiliations:** 1 Department of Psychology, Division for Clinical Psychology, Psychotherapy and Health Psychology, University of Salzburg, Salzburg, Austria; 2 Centre for Cognitive Neuroscience, University of Salzburg, Salzburg, Austria; 3 Department of Psychology, University of Salzburg, Salzburg, Austria

**Keywords:** Ecological momentary assessment, Ambulatory assessment, Re-experiencing, Analogue trauma, Posttraumatic stress disorder, Trauma film paradigm

## Abstract

Intrusive thoughts, images, and their appraisal remain difficult to study despite their clinical relevance. Clinical studies typically used time-based (frequency and distress per observation period), while analogue studies mainly used event-based (report upon occurrence) assessment. A comparison of intrusion frequency, distress appraisal, compliance, and reactivity across different assessments is mostly lacking, particularly with regard to analogue research. Here, intrusions were induced via aversive films and assessed by a smart phone application for 4 days. Three sampling modes were compared by randomizing participants to one of three conditions: either one, or five time-based daily prompts, or event-based assessment. At the end of the study, all participants reported intrusions once again in a retrospective summary assessment. Results indicate that intrusions and their distress decayed over a few days. The three assessments did not differ in intrusion frequency, distress appraisal, compliance (generally high), reactivity (generally low), or retrospective summary assessment. Across groups, the more aversive and arousing participants rated the film clips and the more reactivity to the electronic-diary assessment they reported, the more intrusive memories they had; assessment modes did not differ on this. Thus, no general differences were found between electronic-diary assessment modes for analogue intrusions, giving researchers flexibility for tailoring ecological momentary assessment to specific study aims.

## Introduction

Up to 90 percent of the population experience a traumatic event during lifetime (Kilpatrick et al. 2013), with involuntary spontaneous recollections of the event typically subsiding after several weeks (e.g. Butler et al. 1995; Holmes et al. 2004). These so-called intrusions may occur in healthy individuals but—if continuing with high frequency—also represent a core symptom of posttraumatic stress disorder (PTSD) (American Psychiatric Association 2013). Though, why do some people experience more intrusions after traumatic events than others? Ehlers and Clark (2000) suggest that PTSD symptomatology manifests particularly when individuals appraise the trauma in a way that leads to a sense of serious current threat. Conceptualizing PTSD from this cognitive perspective, it is the individual’s appraisal of the trauma and subsequently, the appraisal of trauma memories that lies at the core of PTSD symptoms. In order to understand why some people experience more intrusions than others and why some people develop PTSD while others do not, researchers are facing the challenge of capturing intrusions and their appraisal, both of which are relatively fleeting mental events occurring repeatedly in daily life and over extended periods of time.

Reviewing past research, little is known about how to best assess intrusions and their associated appraisal. Three different types of assessment have been used, namely event-based (EB, reporting intrusions upon each occurrence), time-based (TB, signaled entries of intrusions), and retrospective summary assessment (one entry after a couple of days/1 week). Each method has been criticized, with TB possibly triggering intrusions (i.e., *reactivity effects*: one cannot count intrusions without thinking of them, possibly fostering increased occurrence, cf. Shiffman et al. 2008) and retrospective assessment potentially being biased by active reconstruction and memory heuristics (e.g., salient and/or recent experiences are recalled more readily and thus, overestimated retrospectively; Ebner-Priemer and Trull 2009). Furthermore, EB is restricted to subjective reports of compliance (question to the participant at the end of the study whether they missed any entries), whereas objective compliance (number of completed scheduled entries at the end of the study) can only be monitored with TB. Thus, some assessments may cause underreporting whereas others overreporting and they may or may not assess objective compliance.

### Assessing Intrusion Frequency and Their Appraisal in Clinical Studies

Only few clinical studies assessed intrusions EB, like the study by Schönfeld and Ehlers (2017) and the study by Kleim et al. (2013) which combined EB with TB prompts. Most other clinical studies either used retrospective summary (after 1 week, e.g., Hackmann et al. 2004; Speckens et al. 2007) or TB by daily prompts (ranging between 2 and 6 daily prompts, depending on study; e.g., Pfaltz et al. 2010, 2013). Differences in the type of assessment mode may be problematic, as a recent study by Kleindienst et al. (2017) showed that TB using one daily prompt led to higher intrusive memory frequency than EB. Furthermore, although Naragon-Gainey et al. (2012) reported no differences between retrospective summary and daily assessment, Priebe et al. (2013) pointed out that they may not always cohere.

In addition to differences in assessment schedule, clinical studies greatly vary in their assessment of how individuals appraise their intrusions. For instance, Hackmann et al. (2004) assessed distress associated with intrusions, whereas others assessed vividness (e.g., Schönfeld and Ehlers 2017) or general PTSD symptom frequency (e.g., Pfaltz et al. 2010). It is yet unclear to what degree different assessment schedules capture and/or may influence the appraisal of intrusions. Subjective compliance in the assessment of intrusions is generally high. However, objective compliance has either been poor, using 10 random prompts throughout the week (using electronic diaries: Kleim et al. 2013; but note that response window was only 5 min), high, using one daily prompt (Kleindienst et al. 2017), or not reported on (e.g., using electronic diaries: Priebe et al. 2013; 30-day later retrospective assessment:; Naragon-Gainey et al. 2012). Those discrepancies are in line with a review by Shiffman et al. (2008), pointing to a range of 50–90% objective compliance across various clinical studies. As described by Trull and Ebner-Priemer (2013), participants’ compliance depends, among other things, on the intrusiveness and burden of the diary protocol; thus, compliance will likely drop with the number of prompts and length of study period. In addition, allowing subjects to more easily incorporate the e-diary assessment into their daily lives increases “livability” and thus, increases compliance (Hufford and Shields 2002).

### Assessing Intrusion Frequency and Their Appraisal in Analogue Studies

To assess intrusions in non-clinical populations, intrusive memories are typically induced by the trauma film paradigm—a trauma analogue. Due to the lesser intensity of the “trauma”, analogue intrusions may be conceptualized as particularly fleeting cognitive events, making assessment of their frequency and appraisal especially challenging. Past analogue trauma studies mainly used EB paper diaries (e.g., Halligan et al. 2002; James et al. 2016b, a for review) Tabrizi and Jansson 2016; see), one study used EB e-diary combined with two daily prompts (Streb et al. 2016), few studies used e-diaries with prompted entries (e.g., Malik et al. 2014), and few studies used once a day desktop computer assessment (e.g., Das et al. 2016; Kamboj et al. 2014). Thus, to our knowledge, no analogue study compared e-diary to retrospective summary assessment. Furthermore, compared to clinical studies, most analogue studies assessed appraisal systematically by asking for the distress associated with the intrusions. However, no analogue study has yet compared different sampling modes on intrusion frequency and distress appraisal.

Little is known about objective and subjective compliance in analogue studies assessing intrusive memories. Although Malik et al. (2014) reported about 75% completed prompts, most analogue studies use EB designs, restricting researchers to subjective compliance measures. However, subjective compliance is rarely assessed, though, the few studies that assessed it point to generally high compliance (e.g., Măirean and Ceobanu 2016).

As of now, except for the clinical study by Kleindienst et al. (2017) that compared TB using one daily prompt to EB, no other and particularly no analogue study compared several TB entries per day to one TB entry in the evening, compared TB to EB sampling, and compared different e-diary assessments to retrospective summary assessment. In addition, the relationship between different sampling methods (TB, EB) and retrospective summary assessment has yet to be studied: will one sampling method be closer to retrospective summary assessment then another? Does distress appraisal differ between different sampling methods?

### The Present Study

The present study set out to test three different sampling modes against each other in terms of analogue intrusion frequency and distress appraisal, reactivity, and compliance. Intrusions and distress appraisal were assessed 4 days following an analogue trauma using either event-based (EB), time-based once-a-day in the evening (TB1), or time-based five times per day (TB5) e-diaries. All participants rated their overall intrusion frequency and intrusion distress level retrospectively at the end of this diary assessment, similarly to clinical studies using once-a-week retrospective summary assessment.

In terms of frequency, we expected EB to be highly accurate relative to TB, as prompts as potential triggers of additional intrusions do not occur (least reactivity effects) and the immediate report minimizes retrospective biases. Furthermore, we expected TB5 to result in high reactivity and thus, more intrusions, compared to TB1 and EB. In addition, we tentatively expected that the more retrospective assessment modes (TB1, and to a lesser degree, TB5) would be characterized by higher level of intrusion distress due to memory biases leading to exaggerated reports compared to EB (Ebner-Priemer and Trull 2009). Moreover, we expected subjective compliance to be highest in EB, as diary-entry is not bound to specific time-frames, and lowest in TB5 due to the burden of the protocol and decreased “livability”; we expected actual compliance to be lowest in TB1 because of a high chance to miss a large proportion of overall entries with just one missed entry (vs. TB5). Due to retrospective overreporting, we expected retrospective summary compared to e-diary assessment to be linked to more intrusion frequency and distress; correspondence between retrospective summary assessment and diary assessment may be highest in TB1 (mostly because of the ease of remembering all four previous entries), compared to TB5 and EB. We additionally expected high aversiveness and arousal film ratings and high reactivity to the e-diary assessment to be related to more intrusive memories and distress across all groups.

## Method

### Participants

Seventy-eight healthy women (aged between 18 and 35 years, *M* = 23.26, *SD* = 3.63) were recruited at the University of Salzburg and a local job portal. Exclusion criteria were current mental or neurological disorders, psychiatric medication, a history of psychological trauma, and consumption of violent films above average (more than three times a week). The study was approved by the local ethics committee. Participants gave written informed consent and were compensated with either course credit or a payment of 20 Euros.

### Procedure

During the laboratory session scheduled in the afternoon on day one, participants were seated on a chair placed 60 cm in front of a 24″ full-HD monitor. They filled out the General Depression Scale (ADS-L; German version by Hautzinger and Bailer 1993) and the State-Trait Anxiety Inventory (STAI, German version by Laux et al. 1981), followed by two exemplary Visual Analogue Scale (VAS) ratings and the trauma film. The film consisted of three different scenes (25 s each), which were repeated three times in pseudo-random order (programmed in E-Prime 2.0; Psychology Software Tools, Inc., Pittsburgh, PA); total length was 6.5 min. The film scenes contained severe interpersonal violence and the immediate aftermath of a bloody murder (film A scene from “Antichrist”, 2009, directed by Lars von Trier; film B: scene from “Hostel”, 2005, directed by Eli Roth; film C: scene from “Scar”, 2007, directed by Jed Weintrob; see Wegerer et al. 2013, 2014). Participants were instructed to report intrusive memories of the scenes on the day of film viewing and the following 3 days via an e-diary smartphone application. To compare different sampling modalities, participants were randomly assigned to one of three diary groups: the EB group entered intrusive memories immediately after their occurrence and could postpone their entry, in exceptional cases (e.g., when driving a car). The TB5 group received five signaled prompts per day at 9 am and 12, 3, 6, 9 pm, whereas the TB1 group was prompted at 9 pm only (asking participants to report the number of intrusions since their last entry). To avoid back-filling, each alert allowed a 1-h time frame for completion. At the end of the study, participants completed an online retrospective summary assessment on their computers at home, assessing total number of intrusions since the laboratory session, compliance, and reactivity.

### Measures

#### Emotional Reactions to the Trauma Film

Emotional reactions to the trauma film were rated on a VAS (from 0 = “*not at all*” to 100 = “*extremely*”) after film viewing, in terms of valence, arousal, distress, fear, and disgust.

#### Intrusive Memories

Participants were instructed to fill out a smartphone application during the following 4 days (total of 3.5 days, including ‘half day’ following the laboratory session). Intrusive memories were defined as *“memories about the aversive film clips, which could be images, sounds or thoughts about the film, but also as recurring thoughts or feelings that had been present during watching”* (c.f. Intrusion Memory Questionnaire, IMQ; Ehring et al. 2009; Zetsche et al. 2009). For EB assessment, participants were asked to fill out the smartphone app whenever an intrusive memory occurred; in case participants were not able to complete the questionnaire right away, they had the option to postpone their entry. If so, they could indicate this in the application and note down the exact time when the intrusive memory had occurred. For TB5 assessment, participants were instructed that they would be prompted by a sound five times per day to report the total number of intrusive memories since their last entry (participants were not informed about the exact timing of prompts); they were allowed to postpone this entry for up to 1 h if circumstances did not allow immediate entry. For TB1, participants were instructed to fill out the smartphone application once a day at 9 pm, prompted by sound; participants indicated the number of intrusions according to six time-windows (up to 9 am, 9 am–12 pm, 12 pm–3 pm, 3 pm–6 pm, 6 pm–9pm, after 9pm). Participants should report involuntary memories only and no deliberate recall (e.g., recall directly prompted by the diary questions); participants were further instructed to report intrusions during the night (e.g., during wake times or dreams) as part of their first entry on the following day. Participants should also report the distress associated with the intrusions (VAS slider from 0 “*not at all*” to 100 “*extremely distressing*”). If participants did not report any intrusions in the TB diary entry, this score was set to 0; furthermore, if the EB group did not report any intrusion for a respective day, intrusion frequency and distress were set to zero for this day. Intrusions were assessed via a customized e-diary smartphone application called PsyDiary especially designed in collaboration with the Smart Health Check research group at the department of MultiMediaTechnology of the Salzburg University of Applied Sciences (cf. Reichenberger et al. 2016). Supported platforms are Android and iOS with ecological momentary assessment (EMA) questions being accessed and defined via Limesurvey (Schmitz 2015). To familiarize each participant with the smartphone application, participants completed a demo questionnaire together with the experimenter.

#### Compliance and Reactivity

In TB, objective compliance was measured via the percentage of missed diary prompts in relation to overall diary prompts; in EB, participants indicated at each diary entry whether the respective intrusion had occurred right before or in the immediate past. Subjective compliance was assessed online during post-study assessment with the question “Please indicate how the following statement applies to you: I have often been unable/forgotten to enter my involuntary memories into the diary” (on a visual-analogue scale from “*not at all*” = 0 to “*very often*” = 100; cf. Holmes et al. 2004). The total number of reported diary intrusions was also considered an objective index of possible reactivity effects resulting in more intrusions in one versus the other condition(s). To assess self-reported reactivity effects, participants completed an adapted 13-item version of the questionnaire by Ebner (2004) during post-study assessment (example questions: “How much do you think that filling out the smartphone app increased your spontaneous memories to the aversive film scenes?”; “How much did you feel that filling out the smartphone app disturbed your daily life?”, from (“*not at all*” = 0 to “*very often*” = 100); α = 0.74; items were averaged to obtain a total score of subjective reactivity).

### Statistical Analyses

Of the 78 participants, four were excluded due to technical e-diary problems, one terminated participation early. Therefore, 73 participants were included in the final analyses (EB, *N* = 25; TB1, *N* = 25; TB5, *N* = 23).

Like in the study by Kleindienst et al. (2017), non-parametric testing was used for intrusion data since the number of reported intrusive memories was right-skewed and could not be normalized by any transformation. The Kruskal–Wallis test was used to test for differences between sampling modes in intrusion frequency and distress appraisal, as well as self-reported compliance and reactivity. The sign test was used to compare correspondence between e-diary and retrospective summary assessment between groups. To test for group differences between e-diary and retrospective summary assessment, we first computed a difference score and then compared it between groups. The time courses of intrusions and distress appraisals were computed by comparing successive days with each other using the sign test. This was followed by testing for differences between groups using successive days difference scores. Note that e-diary recording for Day 1 started at about 4 pm (after film viewing). Spearman correlation was used to test for a relationship between reactivity and frequency as well as distress appraisal of intrusions and to test for a relationship between film ratings and frequency as well as distress appraisal of intrusions.

Bayes factors (*BF*) computed in JASP (2017) are reported for main results, allowing interpretations in favor of the H_0_ (no group differences): *BF* < 1/3 can be interpreted as evidence for H_0_, *BF* > 1/3 and < 3 as no evidence to speak of, and *BF* > 3 is evidence for H_1_ (see Dienes 2011). A default Cauchy prior distribution with scale $\sqrt{2}$/2 was used, as implemented in JASP (see Ly et al. 2016).

## Results

No differences between e-diary groups were found on trait anxiety [overall *M* = 37.01, *SD* = 7.54; *F*(2, 70) = 0.12, *p* = .889, *ŋ*^2 ^< 0.01], symptoms of depression [overall *M* = 11.75, *SD* = 7.79; *F*(2, 70) = 0.67, *p* = .516, *ŋ*^2 ^= 0.02], and film scene ratings [overall negative valence, *M* = 83.00, *SD* = 13.10, *F*(2, 70) = 1.37, *p* = .261, *ŋ*^2^ = 0.04; overall arousal, *M* = 60.59, *SD* = 21.63, *F*(2, 66) = 0.79, *p* = .456, *ŋ*^2^ = 0.02; overall distress, *M* = 65.07, *SD* = 21.60, *F*(2, 66) = 0.07, *p* = .934, *ŋ*^2^ < 0.01; fear, *M* = 65.58, *SD* = 23.20, *F*(2, 70) = 0.51, *p* = .065, *ŋ*^2^ = 0.01; overall disgust, *M* = 72.45, *SD* = 17.58, *F*(2, 70) = 1.32, *p* = .273, *ŋ*^2^ = 0.04]. All participants finished testing at around 16:27 (*SD* = 1 h 5 min, median = 16:00) and e-diary groups did not differ [χ^2^(2) = 1.57, *p* = .445].

### Intrusion Frequency Across Sampling Modes and Its Correspondence with Retrospective Summary Assessment

**Fig. 1:**
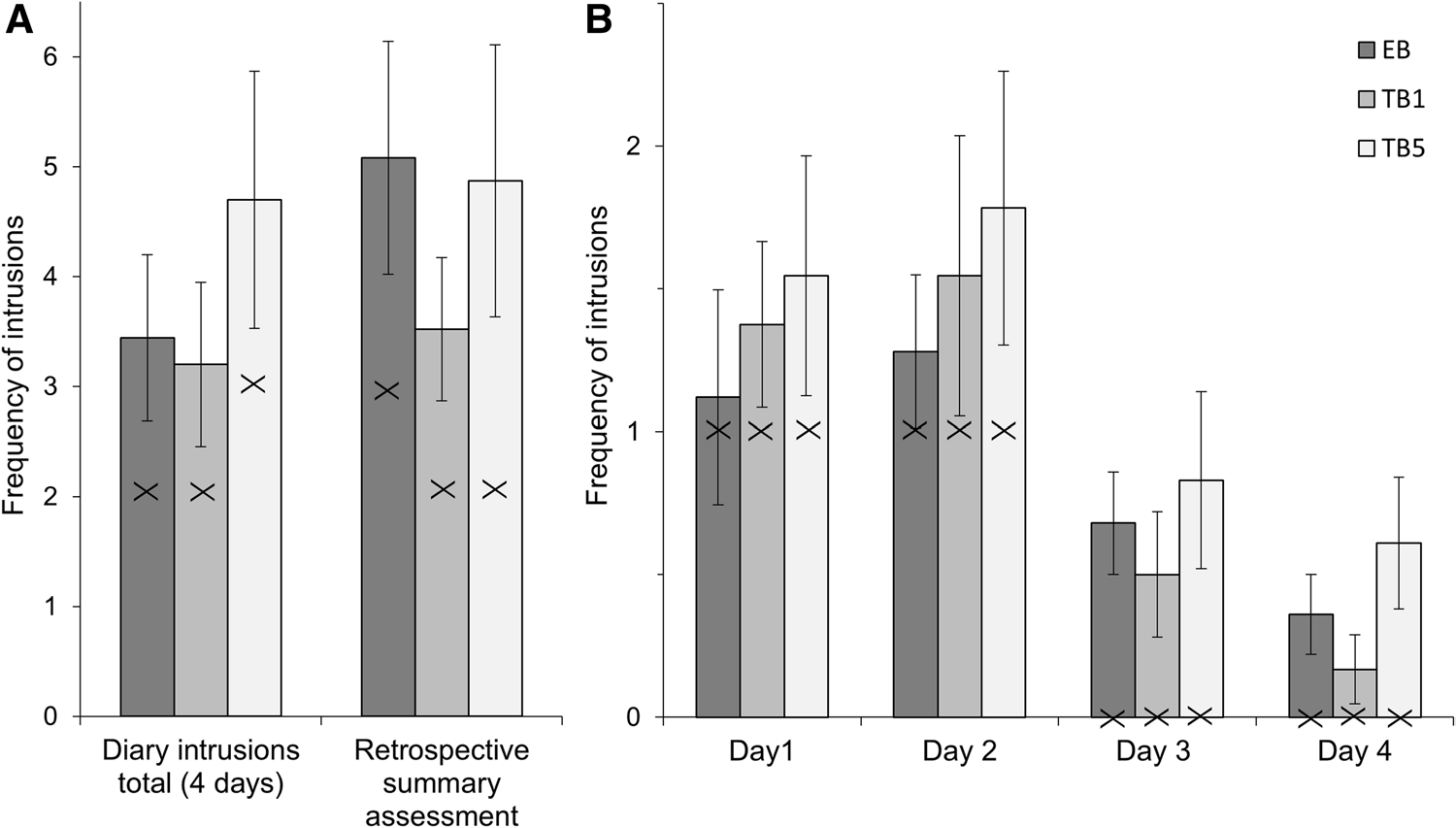
Comparison between e-diary and retrospective summary assessment sum of intrusions (**a**) and the time course of e-diary intrusions (**b**) between the three e-diary assessment groups (means and standard errors, crosses represent the median; *EB* event-based, *TB1* time-based once-a-day in the evening, TB5 = time-based five times per day). Note: e-diary recording for Day 1 started at about 4 pm (after film viewing)

16.0% of participants in EB, 24.0% in TB1 and 21.7% in TB5 reported no intrusions across the 3-day assessment [χ^2^(1) = 0.52, *p* = .771]. Analyses revealed no differences between the three sampling modes regarding the total number of intrusions reported in the e-diary [χ^2^(2) = 0.54, *p* = .764; *BF* = 0.21] as well as during retrospective assessment [χ^2^(2) = 0.88 *p* = .645; *BF* = 0.20] (see Fig. 1a).

Across groups, participants did not report significantly more intrusions by e-diary (Median = 2; Mean = 3.75, *SD* = 4.41, range 0–21) than by retrospective summary assessment (Median = 3; Mean = 4.38, *SD* = 4.93, range 0–23; *z* = − 0.89, *p* = .059; *BF* = 3.89).

Comparing the frequency of intrusive memories over successive days, there was no decrease from day 1 (‘half day’) to day 2 across groups (*z* = 1.19, *p* = .223). Intrusive memories steeply decreased from day 2 to day 3 (*z* = − 0.395, *p* < .001), with a median of zero across all three groups on day three; thus, no difference was found between day 3 and day 4 (*z* = − 1.23, *p* = .201) and no difference between e-diary groups (*p*s ≥ .578; see Fig. 1b).

### Distress Appraisal Across Sampling Modes and Its Correspondence with Retrospective Summary Assessment

**Fig. 2:**
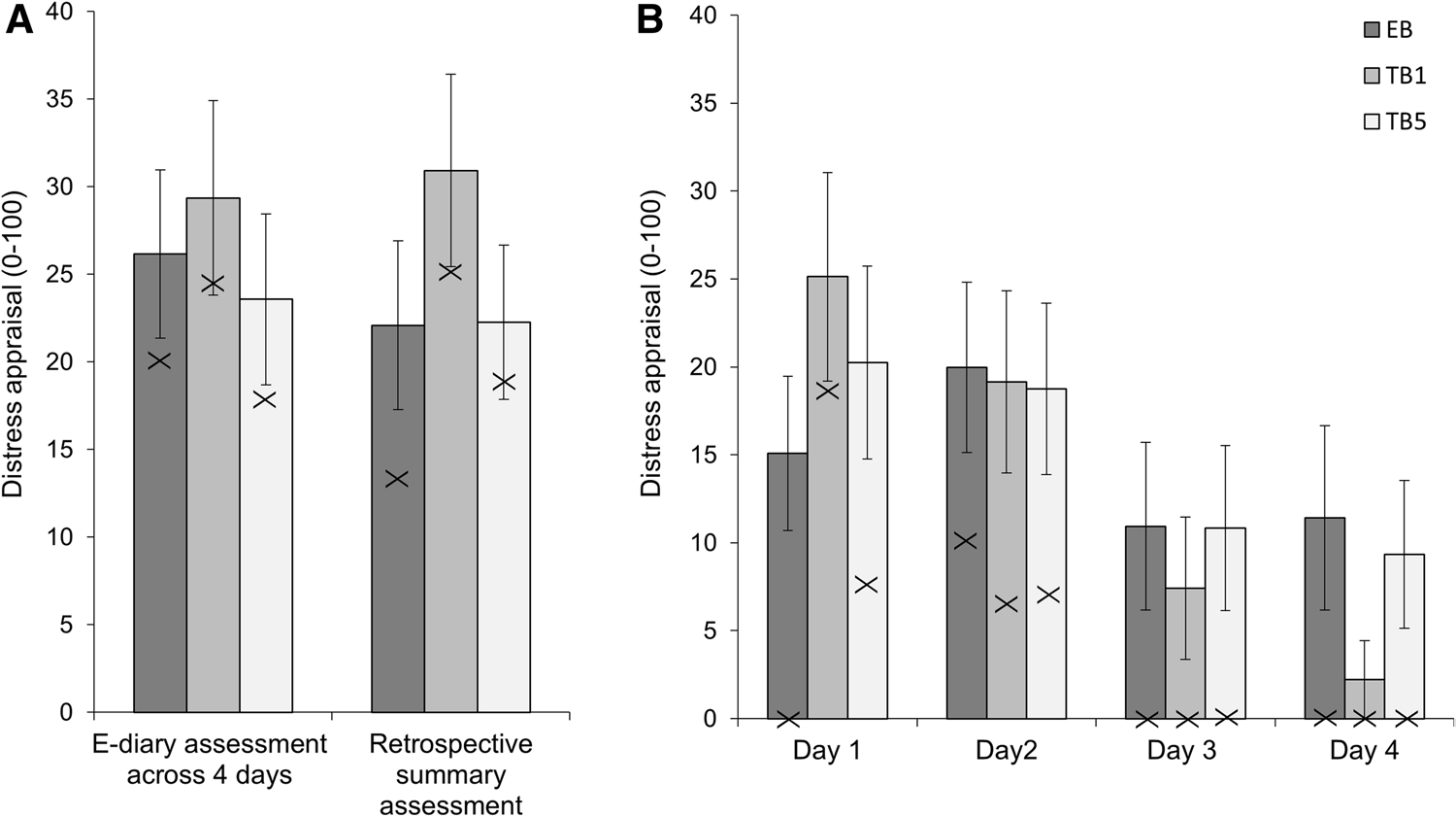
Comparison between e-diary and retrospective summary assessment mean intrusion distress (**a**) and time course of e-diary intrusion distress (**b**) between the three e-diary assessment groups (means and standard errors, crosses represent the median; *EB* event-based, *TB1* time-based once-a-day in the evening, TB5 = time-based five times per day). Note: e-diary recording for Day 1 started at about 4 pm (after film viewing)

To compare e-diary with retrospective distress, a mean e-diary distress score was computed by only counting in days on which participants reported intrusions; for participants who did not report any intrusions across the 3 days, their mean score was set to 0. Analyses revealed no differences between the three sampling modes regarding the average distress appraisal reported in the diary [χ^2^(2) = 0.34, *p* = .845; *BF* = 0.14] as well as during retrospective assessment [χ^2^(2) = 2.00 *p* = .367; *BF* = 0.26]. E-diary (Median = 20.50, Mean = 26.54, *SD* = 25.00, range 0–82) and retrospective summary assessment of distress appraisal (Median = 19, Mean = 25.16, *SD* = 24.43, range 0–88) did not significantly differ [*z* = − 0.66 *p* = .511, *BF* = 0.21; see Fig. 2a).

Comparing the distress appraisal of intrusive memories over successive days, there was no decrease from day 1 to day 2 across groups (*z* = 0.00, *p* = 1.00). However, distress decreased from day 2 to day 3 (*z* = − 3.66, *p* < .001). Across groups, participants reported a median of zero distress on day 3 and thus, no difference in distress was found from day 3 to day 4 (*z* = − 1.54, *p* = .124) and e-diary groups did not differ on this (*p*s ≥ .378; see Fig. 2b).

### Subjective Compliance and Reactivity

Subjective non-compliance (0–100%) did not differ between the three diary groups (EB: Median = 1, Mean = 8.84, *SD* = 17.77, range 0–64; TB1: Median = 6, Mean = 22.48, *SD* = 31.41, range 0–92; TB5: Median = 8, Mean = 10.57, *SD* = 12.59, range 0–52); [χ^2^(2) = 3.32, *p* = .191, *BF* = 0.95], with overall low ratings in terms of missed entries. Self-rated reactivity was also rather low (EB: Median = 26.38, Mean = 26.57, *SD* = 12.05, range 5–48; TB1: Median = 24.92, Mean = 26.17, *SD* = 10.59, range 8–58; TB5: Median = 22.23, Mean = 28.34, *SD* = 13.56, range 11–41) and no group differences were found [χ^2^(2) = 0.12, *p* = .943, *BF* = 0.14].

Reactivity was related to the frequency of intrusive memories (*r*_*s*_ = .25, *p* = .033); although groups did not significantly differ on this, there was a tendency for reactivity to be most related to the frequency of intrusive memories in TB5 (*r*_*s*_ = .40, *p* = .060). Furthermore, reactivity was also related to distress appraisal (*r*_*s*_ = .280 *p* = .016); although groups did not significantly differ on this, there was a tendency for reactivity to be most related to the distress appraisal of intrusions in TB5 (*r*_*s*_ = .48 *p* = .020).

### Objective Compliance

The TB1 condition (receiving a total of four signals) missed 16.0% (*SD* = 18.9) of prompts; 48.0% of participants missed one or two signals (52.0% missing none, 32.0% missing 1, 16.0% missing 2). In the TB5 condition (receiving a total of 17 signals), 11.0% (*SD* = 10.5) of prompts were not completed (implying less than two prompts were missed on average), with 47.8% of participants missing between one to two entries (21.7% missing none, 34.8% missing 1, 13.0% missing 2, 13.0% missing 3, 17.4% missing four to six signals). Group comparison revealed that TB1 and TB5 did not differ significantly in the percentage of missed signals [χ^2^(1) = 0.01, *p* = .915; *BF* = 0.48]. Furthermore, in the EB condition, participants could postpone their entry; participants used this function for 21.7% (*SD* = 29.3) of their entries (*N* = 21, as four participants did not report any intrusions and thus, did not make any entry).

### Relationship Between Film Ratings and Intrusive Memories

The more aversive participants rated the films, the more intrusive memories they reported (*r*_*s*_ = .247, *p* = .035); however, film valence ratings were not related to distress (*r*_*s*_ = .199, *p* = .092). The more arousing participants rated the films, the more intrusive memories (*r*_*s*_ = .282, *p* = .019) and distress (*r*_*s*_ = .283, *p* = .018) they reported. Groups did not significantly differ on this; however, there was a tendency for TB1 to show the strongest relationship between arousal ratings and frequency (*r*_*s*_ = .440, *p* = .036) as well as distress (*r*_*s*_ = .529, *p* = .009) and for TB5 between valence ratings and frequency (*r*_*s*_ = .474, *p* = .022).

## Discussion

To our knowledge, this is the first analogue study systematically investigating intrusion frequency and distress appraisal, as well as their trajectories across days as a function of assessment schedule. Intrusions are fleeting cognitive-affective phenomena occurring spontaneously in daily life and thus the issue of reactivity (prompting thoughts during assessment) is a crucial one. We experimentally induced intrusions using the trauma film paradigm, an established method for eliciting analogue intrusions over several days (Holmes et al. 2004; Laposa and Alden 2008). Interestingly, against our expectations, we did not find any differences in intrusion frequency, distress appraisal, reactivity, or compliance between event-based, five-times-a-day, and once-a-day evening assessment. Reactivity effects were rather low and in line with Kleindienst et al. (2017), high reactivity was related to more intrusive memories and more distress appraisal and, descriptively, this tended to be particularly the case in the five-times-a-day group. Subjective and objective compliance were similarly high across all groups. Null-findings for intrusion frequency and distress appraisal, as well as for subjective compliance and reactivity, revealed Bayes factors below 1/3, implying that the evidence statistically supports H_0_ (no group differences).

The present study did not reveal any e-diary group differences in terms of the frequency of intrusive memories. Those findings are contradictory to findings by Kleindienst et al. (2017); investigating intrusions in a clinical PTSD sample, patients reported many more intrusive memories during time-based than during event-based assessment. Those differences between clinical and analogue studies are possibly due to the less severe nature of analogue intrusions, making them less frequent and thus, easier to recall correctly, explaining the null findings in terms of assessment mode. Furthermore, compared to analogue intrusions, the high self-relevance and salience of clinical intrusions may make them more prone to active reconstruction and memory heuristics (particularly overestimation, Ebner-Priemer and Trull 2009). In addition, PTSD patients generally report more depressive symptomatology and anxiety, thus more negative current mood states compared to analogue student samples, which may bias recall of intrusive memories during time-based assessment in favor of overreporting (e.g., Fredrickson 2000). It is yet to investigate if differences in assessment mode also influence the reported distress associated with the intrusions in a clinical sample; the present study did not find any differences in distress appraisal across e-diary groups in an analogue sample.

Across participants, retrospective summary assessment revealed similar intrusion frequency and distress as e-diary assessment. Those findings are somewhat contradictory to past clinical studies that either showed a tendency to overreport retrospectively (30 days later) compared to once-a-day daily assessment (Naragon-Gainey et al. 2012), or that showed more intrusions during e-diary than retrospective assessment (1 week later; Priebe et al. 2013); however, latter findings could be explained by frequent prompting (every 2 h), which possibly induced high reactivity effects (average intrusion frequency during e-diary assessment was 75 intrusions). Present findings suggest that once-a-week assessment is not so different compared to time-based daily assessments in the present study and may therefore be well suited for intrusive memory assessment in analogue studies; however, the validity of retrospective summary assessment in clinical studies is yet in need of further testing.

In line with past trauma film studies (e.g., Butler et al. 1995), the present study could show the viability of assessing analogue intrusions after trauma films, observing an average of 3.5 intrusions during three consecutive days of assessment, with an average distress level of 26.7 on a 0–100 scale. This underscores the ongoing, spontaneous activity of emotional memory after traumatic film viewing and validates the approach. As would be expected and congruent with previous results (see e.g. review of Clark et al. 2015), perceiving analogue trauma as aversive and arousing increased intrusive memory frequency and distress appraisal. Moreover, the present study confirmed that experimentally induced intrusions and distress decay over the course of time and importantly, are short lived and only occur for a few days (Bailey et al. 2011).

In the following, we want to highlight some further advantages and drawbacks of each respective mode that may guide design decisions in future intrusion studies; specifically, each method constitutes a different tradeoff between naturalistic, event-based assessment, experimental control (measures of actual compliance), and participant burden (Ebner-Priemer and Trull 2009; Trull and Ebner-Priemer 2013). Though once-a-day evening assessment might be most efficient for researchers and participants, missing data is most problematic with this type of assessment since any missed entry constitutes a large proportion of data. In addition, retrospective reports even over a 1-day period can be exaggerated and influenced by participants’ current context and mood state, making them somewhat unreliable (e.g., Fredrickson 2000; Kahneman et al. 1993). In addition, retrospective assessment is particularly problematic for unstable processes (e.g., Fredrickson 2000; Perrine and Schroder 2005; Stone et al. 2005), with intrusions having been shown to fluctuate considerably (Johnson et al. 2002).

The possible bias of once-a-day, retrospective assessment regarding fluctuating events may be overcome by more frequent prompting. For instance, recalling intrusions several times per day (instead of once) may reduce retrospective bias and may capture variability across the day; in addition, missing data is less problematic since multi-level statistical models for EMA have been advanced in recent years and can handle interspersed missing entries well (Conner and Mehl 2012). Furthermore, near-threshold intrusions that may be lost using event-based designs and forgotten using once-a-day assessment may be better captured. However, intrusion frequency in PTSD varies between three-to-four intrusions per week and ten intrusions per day (e.g., Hackmann et al. 2004; Naragon-Gainey et al. 2012; Priebe et al. 2013), making it difficult to decide on the appropriate spacing. Furthermore, regarding the trauma film paradigm, intrusion frequency varies between two and six intrusions per week (e.g., Deeprose et al. 2012; Zetsche et al. 2009), with the present study pointing to a median of 2 intrusions (mean: 3.75) for the first four consecutive days. Therefore, five daily prompts may seem too frequent with regard to the present study, however, may be too rare for investigating highly symptomatic clinical populations.

Event-based assessment is disputably the most ecologically valid type of assessment, capturing intrusions and associated appraisals right in the moment when they occur. Remembering intrusions in order to explicitly recall them later during time-based, more retrospective assessment may not always work well, as in PTSD, perceptual memory (closely related to involuntary recall) may be functionally independent of episodic memory (closely related to voluntary recall, Brewin 2014); thus, event-based, compared to time-based assessment may more accurately map onto involuntary trauma memories. However, as we did not find differences between the three EMA sampling modes with regard to the overall frequency and distress of intrusions, the difference between voluntary and involuntary memory may not fully come to play in analogue trauma as participants do not experience strong discordance between perceptual and episodic memory like PTSD patients do (e.g., see theories by Brewin et al. 1996; Ehlers and Clark 2000). Event-based assessment may further be beneficial in reliably assessing the exact intensity of an experience, associated appraisals, triggering events, and variability (see, e.g., Ebner-Priemer et al. 2009).

Although event-based assessment holds many advantages, it has also been criticized for causing an underestimation of symptoms, particularly for less severe ones, as they may not exceed the personal threshold for activating the recording device (Takarangi et al. 2014). Furthermore, when using event-based assessment, researchers cannot measure and ascertain actual compliance. This is particularly relevant in studies of analogue intrusions since a sizeable proportion of participants reports no intrusions at all (e.g. Verwoerd et al. 2011) and this may be due to low compliance. Comparing the present findings to past analogue intrusion studies, most studies used event-based paper-and-pencil diaries. As shown by Stone, Shiffman, Schwartz, Broderick, and Hufford (2002), paper diaries are prone to backfilling and hoarding, with high subjective compliance (~ 91%) and very low objective compliance (~ 11%); though, Green et al. (2006) did not find much difference. Furthermore, carrying around paper diaries may cause high reactivity effects (e.g., see James et al. 2016b: healthy control subjects reported six intrusions over a week), with such diaries being constant reminders of the analogue trauma itself and thereby artificially triggering intrusions; the present study tried to overcome this problem by using participants’ smartphones which participants are accustomed to. Future studies should check for differences using e-diaries compared to paper-and-pencil diaries regarding frequency of intrusions, reactivity, and compliance in this field of research, as we do not know yet if results are comparable.

Lastly, we want to emphasize the importance of not only assessing the frequency of intrusive memories, but also their corresponding level of distress. Spontaneous autobiographical memories are not inherently negative (see Holland and Kensinger 2010), with their appraisal heavily depending on the individual’s interpretation. Appraising intrusions as negative has been repeatedly linked to PTSD development and maintenance (Halligan et al. 2003; Steil and Ehlers 2000) and negative thoughts and feelings related to the trauma are a criterion required for a diagnosis of PTSD with DSM-5 (American Psychiatric Association 2013). Although most analogue studies assessed the level of distress associated with intrusions, some findings are solely interpreted in terms of the frequency of intrusions (e.g., James et al. 2016b). We believe that this approach may restrict generalizability to clinical intrusions. Therefore, future studies should not only consider how to best assess the frequency of intrusions, but also consider how to best assess the distress associated with them and possibly aggregate these two indices into one, clinically more relevant index (e.g., “overall intrusion load” defined by the product of intrusion frequency and intrusion distress, see Rattel et al., submitted).

### Limitations

Although the present study revealed no differences in intrusion reports across e-diary assessment modes and had sufficient power to provide statistical support for absence of these differences using the Bayes approach, we cannot rule out that assessment itself triggered reactivity effects (monitoring of a behavior may by itself increase the behavior) equally across groups (Clemens et al. 2008); however, reactivity was rather low across groups. Ideally, future studies that aim at controlling for reactivity effects should include an experimental condition that completes the retrospective assessment at the end of the study only. Moreover, we can only make claims with regard to maximum 4-day assessment; longer recording periods may reveal increasing differences in intrusion frequency and distress as well as in compliance and reactivity; one may, e.g., expect the higher subject burden in the five-times a day assessment condition to results in reduced long-term compliance in an analogue study. Furthermore, some of the differences in the extant literature reporting on the frequencies of intrusions probably relate to differences in the instructions and definitions given to participants as well as the exact trauma films used; this may restrict generalizability of the present findings. Lastly, the present study tested women only and thus, findings may not generalize to men. As research showed that women access emotional memories faster and more easily than men (e.g., Cahill et al. 2004; Ros and Latorre 2010), this may explain why no assessment mode differences were found between event-based and time-based assessment in a female sample; assessment mode differences may manifest in a male sample.

## Conclusion

To conclude, we could show the viability of assessing analogue intrusions after the trauma film using a smartphone e-diary application and observed a median of two intrusions (mean: 3.75) on average. This underscores the ongoing activity of spontaneous memory retrieval after traumatic film viewing and further validates the trauma film approach. We found that e-diary assessment schedule did neither affect frequency nor distress appraisal of analogue intrusions and did not influence retrospective summary assessment. Although no differences were found, we believe that event-based assessment might be superior, if capturing the involuntary, perceptual memory aspect of intrusions and their subtle mental concomitants is the primary interest. Compared to retrospective summary assessment, it is broadly accepted among EMA methods researchers that this type of assessment is least affected by memory biases. However, if researchers wish to have high experimental control by measuring actual compliance, it may be advisable to use time-based assessment with prompt frequency depending on the expected frequency of intrusions and their stability (c.f. Trull et al. 2015).
